# Facile Preparation of Cellulose Beads with Tunable Graded Pores and High Mechanical Strength

**DOI:** 10.3390/polym16060725

**Published:** 2024-03-07

**Authors:** Ranjv Meng, Lin Liu, Xiuping Su, Wenli Gong, Xiaolei Luo, Huiying Gao

**Affiliations:** 1 School of Fashion Design, Jiaxing Vocational Technical College, Jiaxing 314036, China13567307272@163.com (H.G.); 2 School of Materials Science and Engineering, Zhejiang Sci-Tech University, Hangzhou 310018, China; 15757335629@163.com (W.G.); ambious_skyler@163.com (X.L.); 3 Key Laboratory of Functional Fibers and Intelligent Textiles, Shaoxing University Yuanpei College, Shaoxing 312000, China; suxiuping@usx.edu.cn

**Keywords:** cellulose beads, tunable porous, core–shell structure, hierarchical porous, coagulation bath

## Abstract

Cellulose-based hierarchical porous beads exhibit significant application potential in adsorption and separation systems due to their degradation and biocompatibility. However, the current fabrications of cellulose beads show poor mechanical properties and a difficult-to-regulate hierarchical porous structure, reducing their lifespan of use and limiting their application in fine separation. Here, we reported the facile creep–drop method to prepare cellulose beads that enabled systemic regulation of the macro-size, micropore structures, and mechanical properties by optimizing injection nozzle diameter, the composition of the coagulation bath, the temperature of the coagulation bath, and cellulose concentration. Notably, during the molding process, the H_2_SO_4_-Na_2_SO_4_ composite solidification bath endowed cellulose beads with a dense shell layer and a loose core layer, which achieved the integration of mechanical properties and high porosity. The cellulose beads exhibited high porosity (93.38–96.18%) and high sphericity (86.78–94.44%) by modulating the shell thickness of the cellulose beads. In particular, the cellulose beads exhibited excellent mechanical properties with a high compressive strength of 544.24 kPa at a 5% cellulose concentration. It is expected that these cellulose beads with tunable microstructures can realize their potential for applications in the fields of wastewater treatment, chemical engineering, bioengineering, medicine, and pharmaceuticals.

## 1. Introduction

Porous beads, possessing spherical particles, a uniform pore structure, a good functional surface, and a controllable size, can match different application scenarios in adsorption [1], separation [2], chromatography packing [3], catalyst support [4], drug delivery [5], drug release [6], etc. Normally, porous beads have been made from inorganic or organic compounds, such as silica [7], carbon [8], cellulose [9], chitosan [10], alginate [11], polyacrylonitrile [12], etc. In comparison with other materials, cellulose is more attractive as a green advanced material due to its abundant source, renewability, non-toxicity, environmental friendliness, easy modification, and low cost [13,14]. However, due to the high degree of polymerization and strong hydrogen bonding, cellulose shows poor solubility in conventional solvents, resulting in low mechanical properties of regenerated cellulose beads [15,16].

Therefore, a lot of effort is spent on dissolving cellulose and improving the mechanical properties of cellulose beads. There are several ways to dissolve cellulose, such as the early viscose process, cellulose carbamate process, recent aqueous complexing agents, alkaline solution system, and non-aqueous system [17]. Unfortunately, most dissolution methods inevitably come with the use of toxic or expensive reagents such as N-dimethylacetamide, ammonium fluoride/dimethyl sulfoxide, and LiClO_4_, which are not conducive to environmental protection [18,19,20]. The low-temperature alkaline–urea system is currently a promising method for dissolving cellulose by disrupting the intramolecular and intermolecular hydrogen bonds of cellulose molecules, which possesses the advantages of a great solubilization effect, low energy consumption, and low solvent cost [21]. At present, the NaOH–urea system has been applied to the preparation of regenerated cellulose membranes [22], aerogels [23], fibers [24], and porous materials [25,26], thus providing a highly promising approach for the preparation of high-performance regenerated cellulose bead materials.

In terms of the preparation of cellulose beads, the primary methods include drop-in phase separation, emulsion dispersion, high-pressure electrostatic jetting, the microfluidic method, etc. [27,28]. The drop-in phase separation method is one of the simplest and continuous methods to achieve the large-scale production of spherical polymers [29], which is capable of controllably producing spherical substances of different sizes. Compared with other methods, the drop-in phase separation method improves the spherulization efficiency, reduces the preparation cost, decreases the use of organic solvents, and reduces environmental pollution [30]. During the process of preparing porous cellulose beads by drop-in phase separation, the cellulose solution is extruded from the nozzle and dripped into the coagulation bath, and the exchange of solvent and non-solvent promotes the regeneration of the drops by condensation from the outside to the inside to form the porous cellulose beads, whose physical properties and structure depend on the parameters of the preparation process [31]. However, current studies lack dimensional control and rarely discuss the laws of mechanical properties, leading to unclear mechanisms for regulating the structure and properties. On the other hand, since cellulose beads cannot effectively rebuild the three-dimensional hydrogen bonding network in a solidification bath, thus forming a loose network structure, current cellulose beads tend to exhibit poor mechanical properties. In summary, developing cellulose beads with excellent mechanical properties and tunable structures remains an important challenge.

In this study, we developed a simple and accurate method to prepare cellulose beads by using alkaline urea as the solvent and H_2_SO_4_/Na_2_SO_4_ as the coagulation bath. The prepared cellulose beads not only had high porosity and great sphericity but also possessed a core–shell structure: the surface was a dense shell-like structure formed by the deposition of cellulose molecules, and the inside was composed of a three-dimensional network structure of cellulose molecular chains. By investigating the effects of different cellulose concentrations, coagulation bath concentrations, and temperatures on the performance of the cellulose beads, it was found that the cellulose beads of 5% concentration showed excellent mechanical properties with a compressive strength of 544.24 kPa. Remarkably, we found that the morphology of the cellulose beads could be controlled, which was conducive to the control of the mechanical properties of cellulose beads. This work provides a foundation for the design of cellulose materials and tailoring structures for various applications.

## 2. Materials and Methods

### 2.1. Materials

The cotton fibers were provided by Zhejiang Tiandi Cotton Co., Ltd. (Hangzhou, China), and they were used after vacuum drying at 60 °C for at least 6 h. Sodium sulfate (H_2_SO_4_), sodium acid (Na_2_SO_4_), sodium hydroxide (NaOH), and urea were obtained from Sinopharm Chemical Co., Ltd. (Shanghai, China). All chemical reagents were analytically pure and were not further purified.

### 2.2. Preparation of Cellulose Beads with Tunable Pore Size and Structure

**Scheme 1:**
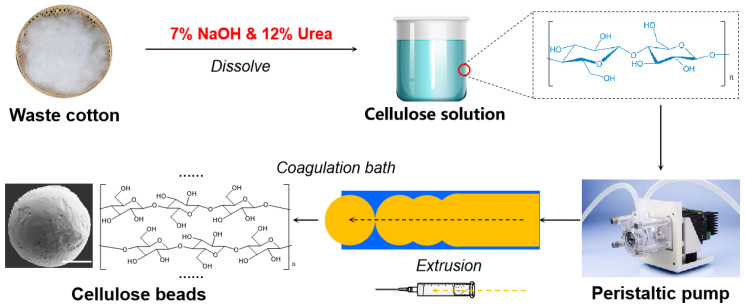
Preparation process of cellulose beads.

As shown in Scheme 1, the preparation of porous cellulose beads was applied from a homogeneous cellulose solution by the dropping method. The process was as follows: a clear homogeneous cellulose solution was formed by dissolving cellulose fibers in a 7% NaOH and 12% urea aqueous solution according to our previous report [32]. Subsequently, the mixed solution was extruded dropwise into a H_2_SO_4_/Na_2_SO_4_ coagulation bath through a peristaltic pump using a syringe needle with a diameter of 1.64 mm, 1.11 mm, 0.52 mm, or 0.11 mm to form the porous cellulose beads. To exclude the impact of other factors (e.g., the surface tension of the coagulant solution, sinking height), the extrusion speed of the mixed solution was maintained at 30 mL/min, with the needle tip kept at a constant distance of 1 cm from the surface of the coagulation bath. All cellulose beads were prepared in 250 mL beakers. The naming of different samples was based on the concentration of different components (the details are in Table 1). To better study the morphology and specific surface area of the cellulose beads and reduce the influence of ice crystals on the internal structure of the balls during freezing, the cellulose balls were instantaneously frozen with liquid nitrogen before freeze-drying and then sent to a Scientz-12N freeze dryer (−80 °C) for drying.

### 2.3. Characterization

#### 2.3.1. Field Emission Scanning Electron Microscope (FE-SEM)

Cellulose beads were brittle-fractured using liquid nitrogen in order to observe their internal structure. Before observation, the cross-section and outside surface of the cellulose bead were gold plated, and then the surface, edge, and internal structures were observed with FE-SEM (JSM-5610, Hitachi, Tokyo, Japan). The acceleration voltage was 3 KV.

#### 2.3.2. Determination of the Weight, Size, and Shape of the Cellulose Beads

Fifty cellulose beads were randomly selected and then gently rubbed with filter paper to dry the excess water on the surface and placed in an oven (60 °C) until the weight was constant. The mass of the dried cellulose beads was weighed, and a Nikon ECLIPSE (TS100, Nikon, Tokyo, Japan) inverted biological microscope was used to measure the size of the cellulose beads. The cellulose beads were randomly selected and placed on a microscope slide to measure the lengths of the long and short axes for calculating the average diameter and sphericity.

#### 2.3.3. Moisture Content

The dry and constant weight method was used to obtain the moisture content. Wet cellulose beads were weighed at a certain amount M_0_ (g) and then placed in an oven (60 °C) to dry until the weight was constant. Subsequently, the weight M (g) of dry cellulose beads was measured, and the moisture content (ω, %) was calculated by following Formula (1):

ω = (M_0_ − M) × 100%/M_0_ (1)

#### 2.3.4. Porosity

The pycnometer method was used to measure the wet true density ρ_p_ (g·mL^−1^) of the cellulose beads. The porosity P (%) was calculated by following Formula (2):

P = (ρ_p_ × ω)/ρ_H_ × 100% (2)

where ρ_p_ is the wet true density of cellulose beads, g·mL^−1^. ω is moisture content, %. ρ_H_ is the density of water, 1 g·mL^−1^.

#### 2.3.5. Nitrogen Adsorption Performance

The 3H-2000PS1 BET tester of Beijing Best Analytical Instrument Research Institute (3H-2000PS1, BeiShiDe, Beijing, China) was used to test the specific surface area of cellulose beads. The samples were first degassed for 2 h at 130 °C, then nitrogen was used as the adsorption medium to determine the equilibrium adsorption capacity of nitrogen at different working pressures, and finally, the specific surface area of the sample was obtained by the BET adsorption equation.

#### 2.3.6. Mechanical Properties

The universal material testing machine (5943, INSTRON, Norwood, MA, USA) was used to test the compressive strength of the cellulose beads. The samples were compressed to 5% deformation at a compression speed of 5 mm·min^−1^. After the sample was restored, they were stayed for 30 s and then compressed to 10%. The above steps were repeated until the deformation of the sample reached 60%, and 5 parallel samples were selected for testing.

## 3. Results and Discussion

### 3.1. Size and Shape of the Cellulose Beads

**Figure 1:**
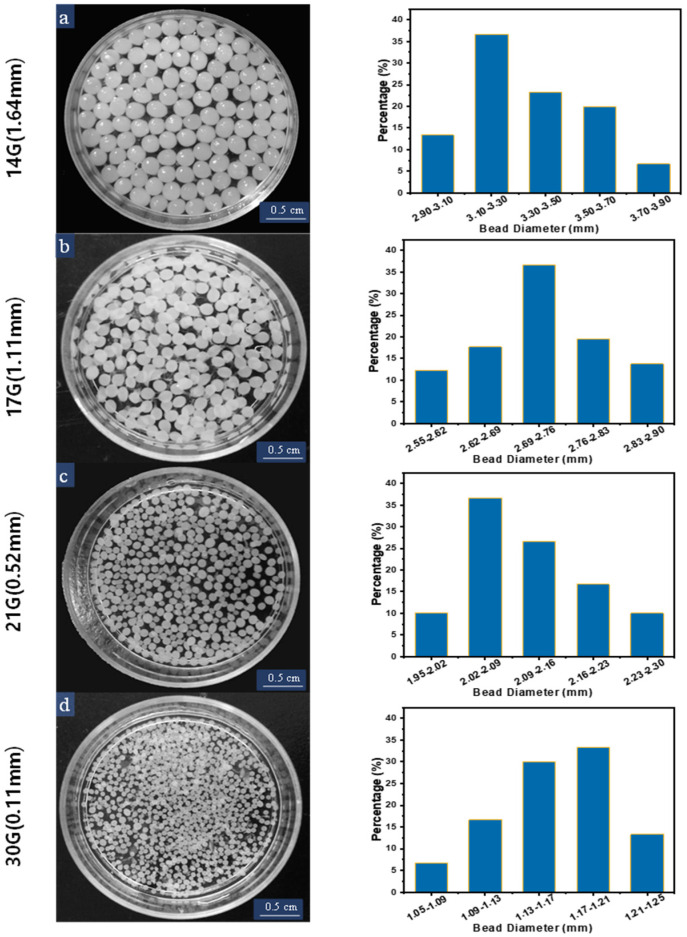
Photos and diameter distribution of the cellulose beads prepared by injection nozzles with different diameters: (**a**) 1.64 mm, (**b**) 1.11mm, (**c**) 0.52 mm, and (**d**) 0.11 mm.

**Figure 2:**
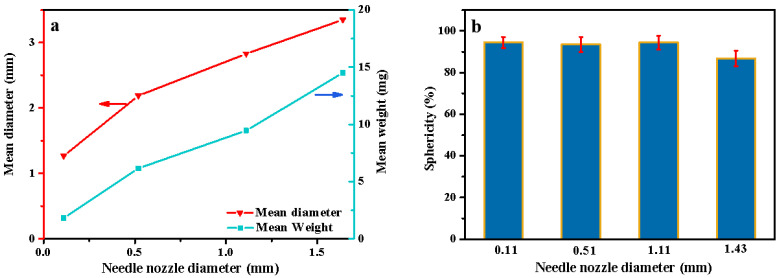
The effect of needle nozzle diameter on (**a**) the mean diameters and weights of cellulose beads and (**b**) sphericity.

Under the combined action of gravity and squeezing force, uniform droplets of different sizes were extruded through needle nozzles with different diameters and then dropped into a H_2_SO_4_/Na_2_SO_4_ coagulation bath to obtain uniform cellulose beads with different sizes. Using this process, the morphology of cellulose beads was explored by adjusting the preparation parameters. The cellulose content, coagulation bath temperature, H_2_SO_4_ and Na_2_SO_4_ concentrations, and extrusion speed were fixed at 4 wt%, 30 °C, 1.5 M L^−1^, 80 g L^−1^, and 30 mL min^−1^. As shown in Figure 1, the formed cellulose beads show a milky white round appearance with a uniform size. The diameter distribution of cellulose beads prepared by using needles with diameters of 1.64 mm, 1.11 mm, 0.52 mm, and 0.11 mm were 2.90–3.90 mm, 2.55–2.90 mm, 1.95–2.30 mm, and 1.05–1.25 mm, respectively. The influences of the diameter of the extrusion nozzle on the average diameter, mass, and sphericity of cellulose beads were studied in detail. As can be seen from Figure 2a, by controlling the diameter of the extrusion nozzle, various cellulose beads with different diameters were prepared, which were 1.27, 2.19, 2.83, and 3.35 mm, respectively. The average weights of the cellulose beads increased with their average diameters, which were 1.82, 6.17, 9.46, and 14.50 mg per bead, respectively. On the other hand, the sphericity of cellulose beads using syringe needles with diameters of 1.64 mm, 1.11 mm, 0.52 mm, and 0.11 mm were 86.78%, 94.35%, 93.49%, and 94.44% (Figure 2b), respectively. All the results showed that the obtained cellulose beads possessed uniform sphericity, thus suggesting the controlled diameters of the cellulose beads. 

### 3.2. Physical Properties of the Porous Cellulose Beads

The cellulose solution was creeped out from the injection nozzle and dropped into the coagulation bath. During the coagulation period of the cellulose droplets, the exchange of the solvent-poor solvent and the non-solvent promoted the extension of the core to continue the growth until the polymer concentration reached its low limitation. When it was too high to be solidified, the coagulation process was completed, and the cellulose beads were formed. Generally, the physical properties and structures of cellulose beads depend on the preparation process parameters, so cellulose beads with different pore structures can be obtained by adjusting the preparation process parameters for application in different fields. In this study, cellulose concentrations and coagulation bath conditions (temperature and H_2_SO_4_ and Na_2_SO_4_ concentrations) were tuned to explore their effects on the physical properties of cellulose beads. The preparation conditions and physical properties of all cellulose samples are summarized in Table 1. To determine the effect of cellulose concentration and coagulation bath conditions on the microstructure of the cellulose beads, the porosity, water content, and specific surface area (SSA) of the cellulose beads were tested and characterized.

The samples in the first group (CBC-3, CBC-4, and CBC-5) were prepared by adjusting the concentration of the cellulose solution. As shown in Table 1, the porosity and water content of cellulose beads decreased with the concentration of the cellulose solution, but the SSA increased from 87.17 to 99.87 m^2^ g^−1^. The samples in the second group (CBA-0.5, CBA-1, CBA-1.5, and CBA-2) were prepared by adjusting the concentration of H_2_SO_4_ in the coagulation bath. It can be seen from the data that the concentration of H_2_SO_4_ mainly affected the SSA: the SSA was only 64.66 m^2^ g^−1^ when the H_2_SO_4_ concentration was 0.5 M L^−1^, and the SSA increased to 97.34 m^2^ g^−1^ when the concentration reached to 1.5 M L^−1^. The third group samples, including CBS-0, CBS-4, CBS-8, and CBS-12, were prepared by adjusting the Na_2_SO_4_ concentration in the coagulation bath, and the effect was similar to the H_2_SO_4_ concentration. The fourth group samples, including CBT-0, CBT-15, CBT-30, and CBT-45, were prepared under different reaction temperatures. The temperature showed a weak influence on the porosity and water content of the cellulose beads, but the SSA was relatively large at 30 °C. When the temperature reached up to 45 °C, the cellulose beads showed the smallest SSA of 58.66 m^2^ g^−1^. In general, the concentration of the cellulose solution had a bigger influence on the porosity, water content, and SSA of the cellulose beads. The higher the concentration, the lower the porosity and water content, and the higher the SSA. The concentration of sulfuric acid, the concentration of sodium sulfate, and the reaction temperature in the coagulation bath mainly affected the SSA of the cellulose beads, and a large SSA could only be obtained within an appropriate range.

### 3.3. Effects of Different Reaction Conditions on the Morphology and Core–Shell Structure of the Cellulose Beads

**Figure 3:**
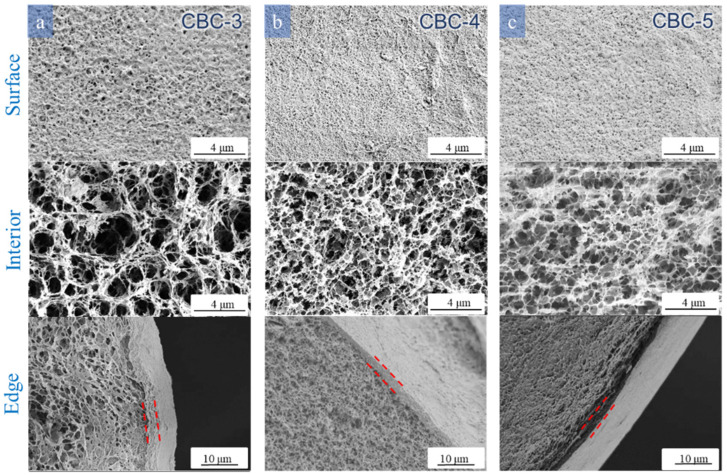
SEM images of the surface, edges, and interior of the cellulose beads prepared with different cellulose solution concentrations: (**a**) 3 wt%, (**b**) 4 wt%, and (**c**) 5 wt% cellulose concentration. The coagulation bath was 1.5 M L^−1^ H_2_SO_4_/80 g L^−1^ Na_2_SO_4_ at 30 °C.

**Figure 4:**
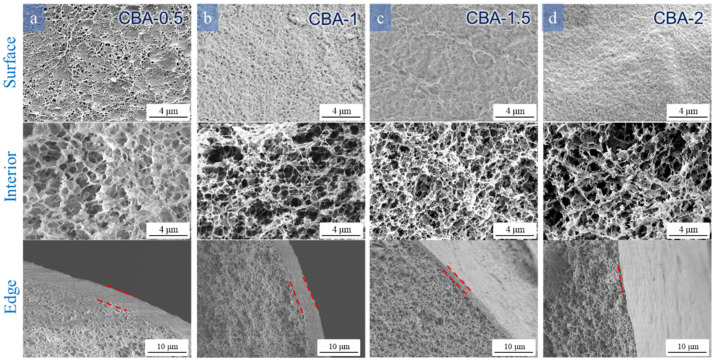
SEM images of the surface, edge, and interior of the cellulose beads prepared with different H_2_SO_4_ concentrations: 4 wt% cellulose concentration coagulated into (**a**) 0.5, (**b**) 1, (**c**) 1.5, and (**d**) 2 M L^−1^ H_2_SO_4_ with 80 g L^−1^ Na_2_SO_4_ and 30 °C.

**Figure 5:**
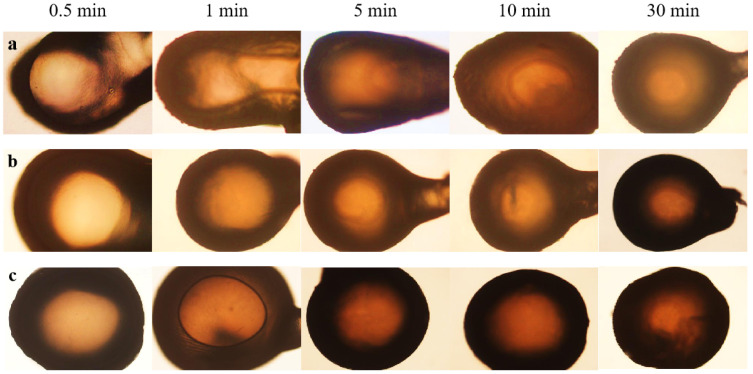
Photos of the cellulose beads prepared with different H_2_SO_4_ concentrations: (**a**) 0.5, (**b**) 1.5, and (**c**) 2 M L^−1^ H_2_SO_4_ with 80 g L^−1^ Na_2_SO_4_ and 30 °C.

**Figure 6:**
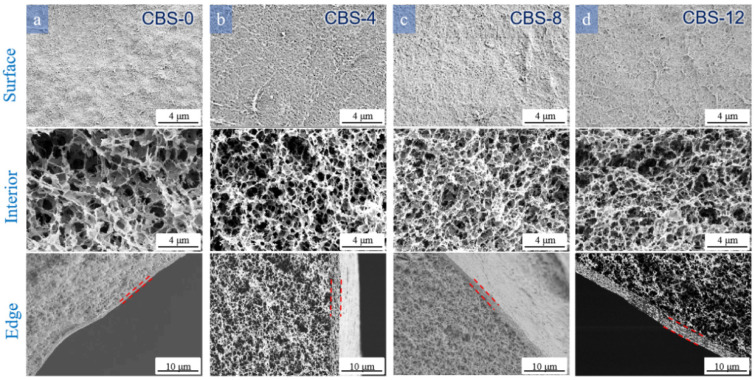
SEM images of the surface, edge, and interior of the cellulose beads prepared with different Na_2_SO_4_ concentrations: 4 wt% cellulose concentration coagulated into (**a**) 0, (**b**) 40, (**c**) 80, and (**d**) 120 g L^−1^ Na_2_SO_4_/1.5 M L^−1^ H_2_SO_4_ at 30 °C.

As shown in Figure 3, Figure 4, Figure 5 and Figure 6, the morphological characteristics of the cellulose beads can be adjusted by changing the cellulose concentration, coagulation bath concentration (H_2_SO_4_/Na_2_SO_4_), and temperature. The shell–core structure played a decisive role in the physical properties. It can be seen from the images that all the cellulose beads showed a well-defined and interconnected 3D network porous structure, and their surfaces were covered by a dense shell. The surfaces of the cellulose beads were thin and dense uniform microporous layers. The internal structures were composed of many holes, each of which was surrounded by thin walls with micropores or mesopores. Of course, the beads produced by different preparation conditions showed some differences in microstructure and macrostructure.

#### 3.3.1. Effect of Cellulose Solution Concentration

Figure 3 shows the effect of different cellulose concentrations on the morphology of cellulose beads. Obviously, as the cellulose concentration increases, the surface of the cellulose beads becomes dense and the size of the micropores decreases. As can be seen from the inside of the cellulose beads, the internal network of the cellulose beads shows a denser structure, respectively. Figure S1 also illustrates this phenomenon. The pore diameter of CBC-3 is larger, and the distribution is wider, mainly distributed in 400~1000 nm. As the cellulose concentration increases, the pore diameter gradually becomes smaller, and the pore diameter of CBC-4 is concentrated in 230~350 nm, and the pore diameter of CBC-5 is concentrated in 100~250 nm. A higher cellulose concentration leads to higher cellulose content in the sample [32,33]. In addition, due to molding under the same coagulation bath conditions, there was no significant difference between the edge thickness of these beads.

#### 3.3.2. Effect of H_2_SO_4_ and Na_2_SO_4_ Concentrations in the Coagulation Bath

Cellulose regeneration is an essential step in the formation of cellulose beads. The coagulant is considered a key factor in the formation process of beads, during which the cellulose solution is regenerated and starts to form beads due to the solvent/non-solvent exchange and subsequent phase separation. Therefore, different coagulation conditions will affect their diffusion and formation process, resulting in regenerated cellulose beads with various morphological characteristics and properties [32]. Figure 4 and Figure 5 show that the H_2_SO_4_ and Na_2_SO_4_ concentrations significantly affect the morphology of the cellulose beads. The kinetics of coagulation can explain this result, which mainly depends on the relative diffusion rate of the solvent from the cellulose droplet to the coagulant and the reverse process, that is, the diffusion of the non-solvent into the cellulose droplet [34]. 

As the concentration of H_2_SO_4_ increased, the surface of the cellulose beads became more compact and smooth (Figure 4). A relatively smooth surface with less pore structure could be observed, and even the pores on the surface of CBA-2 were smaller than those on the surface of CBA-0.5. This indicated that the droplets formed a dense and firm skin after the first contact with more concentrated acid. When the concentration of H_2_SO_4_ increased from 0.5 to 1.5 M L^−1^, there was a denser porous structure in the internal structure of the beads, and the pore size decreased slightly. From Figure S2, it can be seen that the internal pore diameter of CBA-0.5 is larger and the distribution is wider, mainly distributed in 250~700 nm. The pore diameter of CBA-1 is obviously smaller, and the pore diameter is concentrated in 250~390 nm. The increased H_2_SO_4_ concentration significantly enhanced the interdiffusion between the coagulation bath and the cellulose solution and accelerated the process of acid–base neutralization in the internal droplets, which promoted the regeneration of intermolecular hydrogen bonds between the cellulose molecules [35]. However, the strong condensation into 2 M L^−1^ H_2_SO_4_ immediately formed an excessively rigid and dense shell, which hindered the mutual diffusion of the solvent inside the sample and the non-solvent outside the sample. Thus, the inner pores of the cellulose beads had a larger pore size [36]. The pore size of cellulose is significantly wider than that of CBA-1.5, which is mainly distributed between 230 and 350 nm (Figure S2). The internal pore structure was in good agreement with the porosity and BET value of the microbeads (Table 1). On the other hand, a clear core–shell structure could be observed in the cross-sectional image. Coagulation in 0.5 and 1 M L^−1^ H_2_SO_4_ produced a fairly thick skin layer but coagulation in 2 M L^−1^ H_2_SO_4_ formed a relatively thin shell. It could be that the surface of the droplet rapidly regenerated and then immediately formed a very dense and dense skin layer at a high acid concentration. When the concentration of cellulose beads was low, the coagulation process slowed down, and the skin layer of cellulose beads became thick. 

The core–shell formation process of the cellulose beads in a coagulation bath containing different concentrations of H_2_SO_4_ was observed and recorded in detail, as shown in Figure 5. The cellulose solution was dropped into a mixed solution of 0.5, 1, or 2 M L^−1^ H_2_SO_4_ and 80 g L^−1^ Na_2_SO_4_ and taken out at 0.5, 1, 5, 10, and 30 min; the coagulation process was observed under a microscope. The darker areas represent the regenerated cellulose beads, while the bright areas represent the cellulose solution without solidification. At 0.5 min, the edges of the three samples were regenerated, and the central part was still the cellulose solution. After 30 min, the cellulose solution inside the droplet gradually completed regeneration and shrank into spherical beads. From the regeneration process of the sample and the size of the droplet, it could be seen that the coagulation rate of the cellulose beads in the 0.5 M L^−1^ H_2_SO_4_ reaction environment was the smallest, and the sample processed by 2 M L^−1^ H_2_SO_4_ was significantly smaller than the other two samples. This showed that the higher concentration of H_2_SO_4_ tended to cause a denser shell of the cellulose beads. However, at 10 min, the 2 M L^−1^ H_2_SO_4_ sample had more cellulose solution than the 1.5 M L^−1^ H_2_SO_4_ sample, which showed that the dense shell affected the solution exchange between the inside and the outside and hindered the regeneration of the internal cellulose solution.

Figure 6 shows the effect of different doses of Na_2_SO_4_ on the morphology of the cellulose beads. CBS-0 showed a loose and porous internal structure, which is also illustrated by Figure S3, and the edge thickness was thinner. This is because sulfuric acid could be fully ionized without the presence of Na_2_SO_4_. H^+^ ions existed in the coagulation bath, and the exchange rate between the conversion solvent and the non-solvent was faster. The presence of Na_2_SO_4_ promoted the regeneration and formation of beads through a “salting out” mechanism. It is a strong electrolyte and one of the components of the coagulant, which can promote the dehydration and formation of cellulose droplets [35]. On the other hand, absorbing enough Na_2_SO_4_ to H_2_SO_4_ reduced the H^+^ concentration in the coagulation bath and caused the back diffusion rate, thereby contributing to the life of the coagulation bath [37].

#### 3.3.3. Effect of the Temperature of the Coagulation Bath

**Figure 7:**
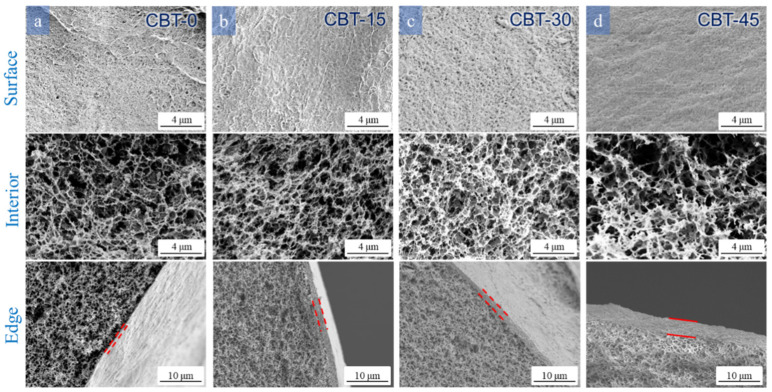
SEM images of the surface, edge, and interior of the cellulose beads prepared with different temperatures: 4 wt% cellulose concentration coagulated into 1.5 M L^−1^ H_2_SO_4_ /80 g L^−1^ Na_2_SO_4_ at (**a**) 0, (**b**) 15, (**c**) 30, and (**d**) 45 °C.

The effect of coagulation bath temperature on the morphological characteristics of the cellulose beads was also studied. The SEM images in Figure 7 showed the effect of different coagulation bath temperatures on the surface, interior, and edges of the beads. It could be seen that raising the temperature of the coagulation bath from 0 °C to 45 °C produced a denser surface and a thicker shell. This phenomenon was closely related to phase transition kinetics. When the cellulose droplets were immersed in the coagulation bath, a shell of droplets was first formed. Subsequently, the coagulation bath of internal droplets depended on interdiffusion and subsequent phase separation. A faster coagulation process occurred with a higher temperature so that the molecular chain of the shell layer was rapidly regenerated to form a thick shell. At the same time, when the coagulation bath temperature was elevated from 0 °C to 30 °C, the internal pores of the cellulose beads became denser. It can be seen from Figure S4 that the internal pore diameter of CBT-0 is larger and the distribution is wider, mainly distributed in 250~700 nm. The pore diameters of CBT-15 and CBT-30 are obviously smaller, with their pore size distributions concentrated in the ranges of 250~390 nm and 230~350 nm, respectively. During the regeneration of the cellulose solution in the coagulation bath, an increase in the temperature of the coagulation bath could enhance the driving force for diffusion between the solvent and the non-solvent. However, when the temperature of the coagulation bath is 45 °C, the internal pores of the cellulose beads become loose, and their pore size distribution changes from 220~350 nm for CBT-30 to 290~410 nm for CBT-45 (Figure S4). It is reasonable that at higher temperatures, faster coagulation might pack cellulose molecules more densely in the shell layer, while at lower temperatures, slower coagulation might fill more cellulose molecules inside. On the other hand, when the temperature of the coagulation bath was from 0 °C to 45 °C, the pores on the surface of the bead gradually became denser, and the thickness of the ball edge gradually increased. In this way, because of the low-temperature conditions, the speed of H^+^ entering the bead becomes slower so that the initial H^+^ and OH^−^ are concentrated on the surface of the cellulose bead for the neutralization reaction, which then produces water, making the surface of the bead have larger holes. The increase in temperature speeds up the diffusion rate, forming a thicker spherical wall.

### 3.4. Mechanical Properties of the Cellulose Beads

**Figure 8:**
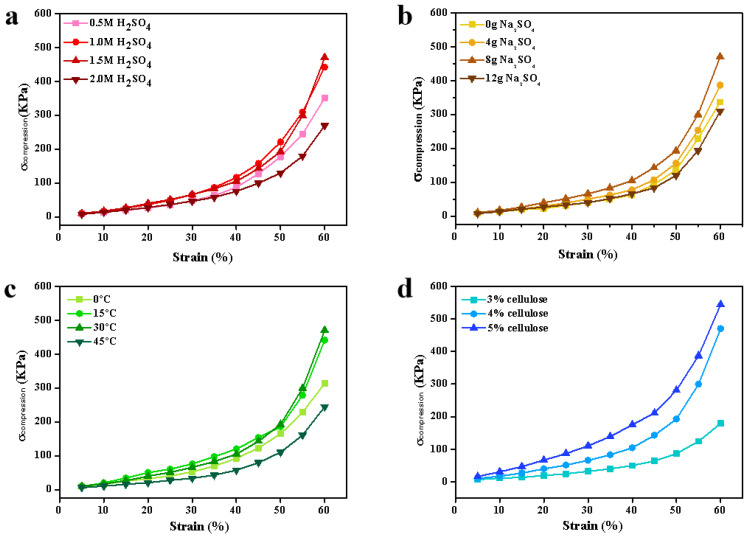
Compression stress–strain curves of the cellulose nanobeads prepared under different preparation parameters: (**a**) cellulose concentration, (**b**) H_2_SO_4_ concentration, (**c**) Na_2_SO_4_ dosage, and (**d**) coagulation bath temperature.

For practical applications, the mechanical properties of a material are extremely important. The compressive stress–strain curves are shown in Figure 8, which indicate that the cellulose beads under different preparation parameters had tunable mechanical properties. Overall, the core–shell structure of the cellulose beads endows the material with a hierarchy at the micro-nano scale [38]. Thus, the compressive strength of the cellulose beads far exceeds the strength reported in the literature (Supplementary Materials). Regarding the cellulose concentration (Figure 8a), the increase in cellulose concentration was beneficial for the strength of the cellulose beads. When the cellulose concentration reached up to 5%, the prepared cellulose beads exhibited the highest compressive strength of 544.27 kPa. This was because the higher concentration of the cellulose solution formed a denser pore structure [39,40] (consistent with Figure 3), which resulted in high mechanical properties. The effects of H_2_SO_4_ concentration and Na_2_SO_4_ dosage in the coagulation bath on mechanical properties were consistent with their effect on the SSA, as shown in Figure 8b. The cellulose beads prepared under the reaction conditions of 1.5 M L^−1^ H_2_SO_4_ and 8 g Na_2_SO_4_ possessed the highest compressive strength of 470.1 kPa, respectively. As shown in Figure 8d, when the temperature of the coagulation bath was 30 °C, the mechanical properties of the cellulose beads were the strongest. The compressive strengths at temperatures of 0 and 15 °C were weak. This was because the lower reaction temperature was not conducive to the formation of cellulose beads, and the excessive temperatures destroyed the stable structure of cellulose beads.

## 4. Conclusions

In this study, a green method was presented to fabricate cellulose beads with a core–shell structure and great mechanical properties. The structure of the cellulose beads was tunable by the concentration of cellulose, the concentration of H_2_SO_4_ and Na_2_SO_4_ in the coagulation bath, and the temperature of the coagulation bath. The concentration of the cellulose solution had a great influence on the porosity, water content, and SSA of the cellulose beads. In terms of mechanical properties, the cellulose beads prepared with a high cellulose concentration (5%) and moderate concentrations of H_2_SO_4_ (1.5 M L^−1^) and Na_2_SO_4_ (80 g L^−1^) had the highest compressive strength. Through the control of these experimental parameters, this work achieved the customization of mechanical properties and apparent morphology of cellulose beads, which showed a huge application potential in wastewater treatment, chemical engineering, bioengineering, medicine, and the pharmaceutical field.

## Figures and Tables

**Table 1:** The codes, preparation conditions, and physical properties of the cellulose beads.

	Preparation Conditions				Porosity (%)	Water Content (%)	SSA (m^2^ g^−1^)	Apparent Density (g/m^3^)
Sample Code	C_cellulose_ (wt%)	Coagulation Condition		T (°C)				
		C_H2SO4_ (M L^−1^)	C_Na2SO4_ (g L^−1^)					
CBC-3	3	1.5	80	30	96.16	94.07	87.17	0.073
CBC-4	4	1.5	80	30	95.85	93.11	97.34	0.078
CBC-5	5	1.5	80	30	93.38	91.36	99.87	0.105
CBA-0.5	4	0.5	80	30	94.85	92.41	64.66	0.076
CBA-1	4	1	80	30	95.21	92.89	94.46	0.071
CBA-1.5	4	1.5	80	30	95.85	93.11	97.34	0.078
CBA-2	4	2	80	30	95.18	92.87	84.84	0.075
CBS-0	4	1.5	0	30	94.67	92.89	60.74	0.071
CBS-4	4	1.5	40	30	95.13	92.98	92.26	0.073
CBS-8	4	1.5	80	30	95.85	93.11	97.34	0.078
CBS-12	4	1.5	120	30	94.45	93.72	80.45	0.079
CBT-0	4	1.5	80	0	95.06	93.27	86.42	0.069
CBT-15	4	1.5	80	15	96.18	92.58	84.93	0.075
CBT-30	4	1.5	80	30	95.85	93.11	97.34	0.078
CBT-45	4	1.5	80	45	93.66	91.04	58.66	0.069

## Data Availability

Data are contained within the article.

## Supplementary Materials

The following supporting information can be downloaded at: https://www.mdpi.com/article/10.3390/polym16060725/s1. Figure S1. Interior pore size distribution of cellulose beads. (a) 3 wt%, (b) 4 wt%, (c) 5 wt% cellulose concentration coagulated into 1.5 M H_2_SO_4_/8 g Na_2_SO_4_ at 30 °C. Figure S2. Interior pore size distribution of cellulose beads. 4 wt% cellulose concentration coagulated into (a) 0.5, (b) 1, (c) 1.5, (d) 2 M H_2_SO_4_/8 g Na_2_SO_4_ at 30 °C. Figure S3. Interior pore size distribution of cellulose beads. 4 wt% cellulose concentration coagulated into 1.5 M H_2_SO_4_ (a) 0, (b) 4, (c) 8, (d) 12 g Na_2_SO_4_ at 30 °C. Figure S4. Interior pore size distribution of cellulose beads. 4% cellulose concentration coagulated into 1.5 M H_2_SO_4_/8 g Na_2_SO_4_ at (a) 0, (b) 15, (c) 30, (d) 45 °C. Figure S5. Comparison of literature on the compressive resistance of cellulose beads [41,42,43,44,45].
