# Supplementary material for: Facile Preparation of Cellulose Beads with Tunable Graded Pores and High Mechanical Strength
Source: Polymers (Basel). 2024 Mar 7;16(6):725. doi: 10.3390/polym16060725 (PMC10975696; doi:10.3390/polym16060725)
Supplement: Supplementary file 1 [file polymers-16-00725-s001.zip › polymers-2785043-supplementary.pdf]

Facile Preparation of Cellulose Beads with Tunable Graded Pores and High Mechanical Strength

Ranjv Meng [1,2], Lin Liu [2,*], Xiuping Su [3], Wenli Gong [2], Xiaolei Luo [2] and Huiying Gao [1]

[1] School of Fashion Design, Jiaxing Vocational Technical College, Jiaxing 314036, China
[2] School of Materials Science and Engineering, Zhejiang Sci-Tech University, Hangzhou 310018, China
[3] Key Laboratory of Functional Fibers and Intelligent Textiles, Shaoxing University Yuanpei College, Shaoxing 312000, China
* Correspondence: linliu@zstu.edu.cn

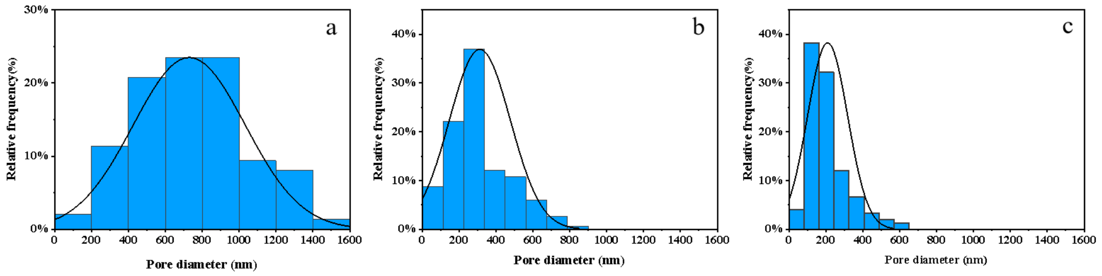

Figure S1. Interior pore size distribution of cellulose beads. (a)3 wt%， (b)4 wt% ,(c) 5 wt% cellulose concentration coagulated into 1.5 M H_2SO_4 /8g Na_2SO_4 at 30◦C.

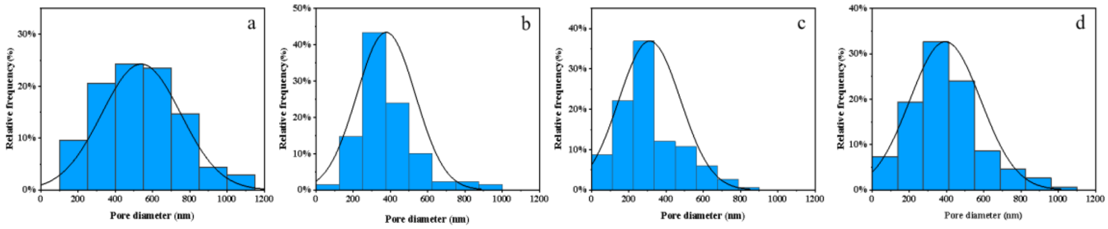

Figure S2. Interior pore size distribution of cellulose beads. 4 wt% cellulose concentration coagulated into (a) 0.5,(b) 1,(c) 1.5,(d) 2M H_2SO_4 /8g Na_2SO_4 at 30°C.

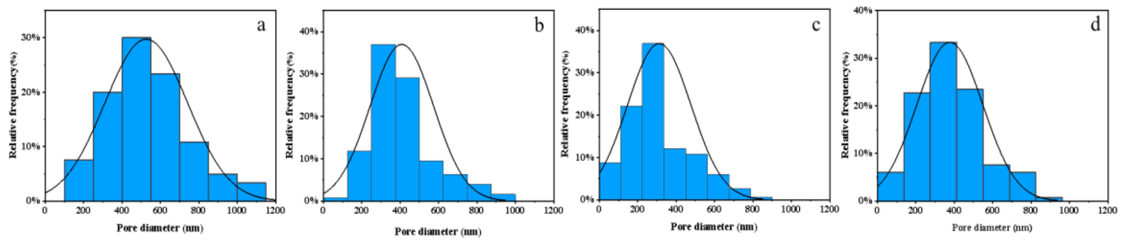

Figure S3. Interior pore size distribution of cellulose beads. 4 wt% cellulose concentration coagulated into 1.5 M H_2SO_4 (a)0, (b)4, (c)8, (d)12g Na_2SO_4 at 30◦C.

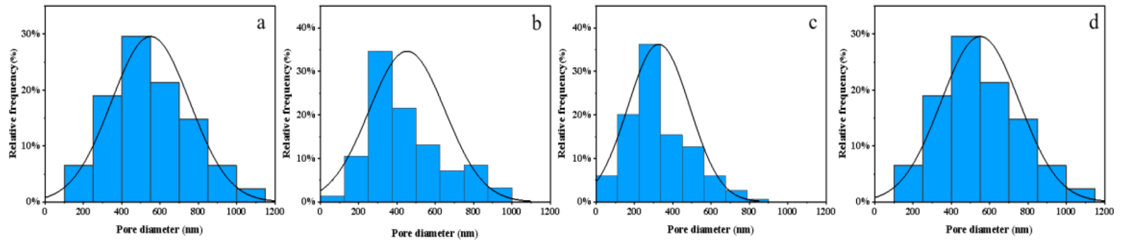

Figure S4. Interior pore size distribution of cellulose beads. 4% cellulose concentration coagulated into 1.5 M H_2SO_4 /8gNa_2SO_4 at (a) 0, (b) 15, (c) 30, (d) 45 °C.

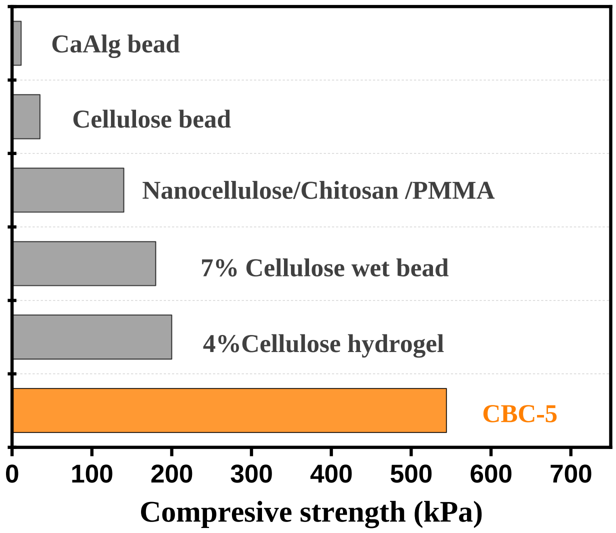

Figure S5. Comparison of literature on the compressive resistance of cellulose beads [1–5].

References

1. Pankongadisak, P.; Ruktanonchai, U.R.; Supaphol, P.; Suwantong, O. Gelatin scaffolds functionalized by silver nanoparticle-containing calcium alginate beads for wound care applications. *Polym. Adv. Technol.* **2017**, *28*, 849–858. https://doi.org/10.1002/pat.3988.
2. Harada, N.; Mitsukami, Y.; Uyama, H. Preparation and characterization of water-swellable hydrogel-forming porous cellulose beads. *Polymer* **2021**, *215*, 123381. https://doi.org/10.1016/j.polymer.2021.123381.
3. Ghavimi, S.A.A.; Lungren, E.S.; Faulkner, T.J.; Josselet, M.A.; Wu, Y.; Sun, Y.; Pfeiffer, F.M.; Goldstein, C.L.; Wan, C.; Ulery, B.D. Inductive co-crosslinking of cellulose nanocrystal/chitosan hydrogels for the treatment of vertebral compression fractures. *Int. J. Biol. Macromol.* **2019**, *130*, 88–98. https://doi.org/10.1016/j.ijbiomac.2019.02.086.
4. Blachechen, L.S.; Fardim, P.; Petri, D.F.S. Multifunctional Cellulose Beads and Their Interaction with Gram Positive Bacteria. *Biomacromolecules* **2014**, *15*, 3440–3448. https://doi.org/10.1021/bm5009876.
5. Cellulose hydrogel with tunable shape and mechanical properties: From rigid cylinder to soft scaffold. *Int. J. Biol. Macromol.* **2018**, *117*, 625–631. https://doi.org/10.1016/j.ijbiomac.2018.05.071.
